# Stereotactic body radiation therapy for post-pulmonary lobectomy isolated lung metastasis of thoracic tumor: survival and side effects

**DOI:** 10.1186/1471-2407-14-719

**Published:** 2014-09-26

**Authors:** Weijie Xiong, Qingfeng Xu, Yong Xu, Changjin Sun, Na Li, Lin Zhou, Yongmei Liu, Xiaojuan Zhou, Yongsheng Wang, Jin Wang, Sen Bai, You Lu, Youling Gong

**Affiliations:** Department of Thoracic Oncology and State Key Laboratory of Biotherapy, Cancer Center, West China Hospital, Sichuan University, Chengdu, 610041 PR China; Radiation Physics Center, Cancer Center, West China Hospital, Sichuan University, Chengdu, 610041 PR China; Department of Radiation Oncology, The Second People’s Hospital of Sichuan Province, Chengdu, 610031 PR China; Department of Oncology, Second Affiliated Hospital of Anhui Medical University, Hefei, 230601 PR China; Chengdu Fifth People’s Hospital and Chengdu Third People’s Hospital, Chengdu, China

**Keywords:** Stereotactic body radiation therapy, Thoracic tumor, Post-lobectomy isolated lung metastasis, Clinical outcomes

## Abstract

**Background:**

Stereotactic body radiation therapy (SBRT) has emerged as an alternative treatment for patients with early stage non-small cell lung cancer (NSCLC) or metastatic pulmonary tumors. However, for isolated lung metastasis (ILM) of thoracic malignances after pulmonary lobectomy, reported outcomes of SBRT have been limited. This study evaluates the role of SBRT in the treatment of such patients.

**Methods:**

A retrospective search of the SBRT database was conducted in three hospitals. The parameters analyzed in the treated patients were local control, progression-free survival (PFS), overall survival (OS), and the treatment-related side-effects.

**Results:**

In total, 23 patients with single ILM after pulmonary lobectomy treated with SBRT were identified and the median follow-up time was 14 months (range: 6.0-47.0 months). Local recurrences were observed in two patients during follow-up and the 1-year local control rate was 91.3%. Median PFS and OS for the studied cohort were 10.0 months [95% confidence interval (CI) 5.1-14.9 months] and 21.0 months (95% CI 11.4-30.6 months), respectively. Acute radiation pneumonitis (RP) of grade 2 or worse was observed in five (21.7%) and three (13.0%) patients, respectively. Other treatment-related toxicities included chest wall pain in one patient (4.3%) and acute esophagitis in two patients (8.7%). By Pearson correlation analysis, the planning target volume (PTV) volume and the volume of the ipsilateral lung exposed to a minimum dose of 5 Gy (IpV_5_) were significantly related to the acute RP of grade 2 or worse in present study (*p* < 0.05). The optimal thresholds of the PTV and IpV_5_ to predict RP of acute grade 2 or worse RP were 59 cm^3^ and 51% respectively, according to the receiver-operating characteristics curve analysis, with sensitivity/specificity of 75.0%/80.0% and 62.5%/80.0%.

**Conclusions:**

SBRT for post-lobectomy ILM was effective and well tolerated. The major reason for disease progression was distant failure but not local recurrence. The PTV and IpV_5_ are potential predictors of acute RP of grade 2 or higher and should be considered in treatment planning for such patients.

## Background

Tumor lung metastasis is one of the most common oncologic problems, and affects a large percentage of patients of cancer despite the histology of the primary tumor. In most cases, widespread metastases are observed. But in certain instances, lung metastasis may exist in isolation. Resection of isolated lung metastasis (ILM) has traditionally been practiced using methods, such as thoracotomy and video-assisted thoracoscopic surgery (VATS) [1–5]. Such approaches have been proved to be effective, achieving a median survival of 35 months, and are associated with generally acceptable morbidity and mortality rates [6]. However, such pulmonary operations have been practiced in salvage treatments for colorectal cancer, breast cancer and other types of tumors, and rarely for thoracic tumor after pulmonary lobectomy.

Hypo-fractionated stereotactic body radiation therapy (SBRT) can deliver high, biologically effective doses to the tumors while minimizing the irradiation dose to the surrounding tissues [7]. Over the decades, SBRT has emerged as an alternative treatment for medically inoperable patients with early-stage non-small cell lung cancer (NSCLC), showing a 5-year survival rate of more than 80% with limited morbidity [8–11]. Even among patients with multiple pulmonary metastases, SBRT has been reported as a safe and effective strategy [12–14]. At present, SBRT is recommended by the National Comprehensive Cancer Network (NCCN) panel as a salvage treatment for patients with ILM [15].

For patients with ILM after pulmonary lobectomy, a few treatment outcomes have been reported to date, including for surgery and SBRT. Therefore, we retrospectively analyzed the clinical outcomes of patients at our institutions with post-lobectomy ILM who were treated with SBRT as a component of their overall treatment regimen.

## Methods

### Patients’ data

ILM in this study was defined as a circular shape ^18^ F-fluorodeoxyglucose positron-emission tomography (FDG-PET) or computed tomography (CT) imaging, without any lobulated signs of original tumor within 3 years after pneumonectomy. We reviewed the records of 268 consecutive patients treated with SBRT for thoracic tumors between October 2009 and December 2013 at the West China Hospital, Second People’s Hospital of Sichuan, and Second Affiliated Hospital of Anhui Medical University. Among these patients we identified 23 who had previously received radical resection of thoracic tumors (including pulmonary lobectomy and systematic lymph node dissection) and who subsequently underwent SBRT to treat the ILM of the ipsilateral or contralateral lung. This retrospective study was carried out with the approval of West China Hospital's ethics committee.

### SBRT treatment

The techniques for patient immobilization and treatment planning have been described in detail in previous reports [16, 17]. In brief, all patients were simulated and treated in stereotactic immobilization body frame with an active breathing control (ABC) device. All CT images (3-mm thickness) of the patients were transferred to and registered in the treatment planning system (Pinnacle^3^, Philips Radiation Oncology Systems, Fitchburg, WI, USA). The gross tumor volume (GTV) was contoured as the identifiable tumor on planning CT in the lung window. The clinical target volume (CTV) enclosed the GTV with a 5-mm margin in all directions. For the planning target volume (PTV), another 5-mm margin was added isotropically to the CTV. The spinal cord, esophagus, bronchus and chest wall were contoured as the organs at-risk (OARs).

Two groups of different doses were given to the PTV, prescribed to the 80 or 90% isodose lines: for small and peripherally located targets, radiation dose was prescribed as 48 Gy/4 fractions or 50 Gy/5 fractions; for targets close proximity to critical structures, radiation dose was prescribed as 56 Gy/7 fractions (Table 1). All fractions were scheduled as three times per week. The dose-volume constraints used for OARs followed the NCCN guidelines [15] and the recommendations from the Radiation Therapy Oncology Group (RTOG) [18]. Plans were generated with five or seven coplanar beams of 6-MV X-rays.

**Table 1:** **Treatment in present study (n = 23)**

Stereotactic body radiation therapy	
***Irradiation dose delivered***	
12 Gy × 4 fractions three times per week	11 (47.8%)
10 Gy × 5 fractions three times per week	9 (39.1%)
8 Gy × 7 fractions three times per week	3 (13.0%)
***Active breathing control***	23 (100%)
***Cone-beam CT guiding***	23 (100%)
***PTV volume (cm*** ^***3***^ ***)***	
Median	48.4
Range	26.0-110.2
***Lung volume (cm*** ^***3***^ ***)***	
Total lung (median)	2301.4
(range)	1983.4-2950.5
Contralateral lung (median)	1373.4
(range)	1255.8-1712.3
Ipsilateral lung (median)	942.2
(range)	786.8-1233.4
**Systematic treatment**	
***Chemotherapy***	
Concurrent chemotherapy	1 (4.3%)
Sequential chemotherapy	14 (60.9%)
***Tyrosine kinase inhibitor*** ^***a***^	3 (13.0%)
***None***	5 (21.7%)

^*a*^: Erlotinib or Gefetinib.

### Treatment assessment and follow-up

Evaluation of treatment response was carried out according to Response Evaluation Criteria in Solid Tumors (RECIST criteria) based on findings from either FDG-PET or CT images [19]. Local recurrence was defined as any re-enlargement of the target if complete response (CR) had not been reached after SBRT or re-appearance of the target if CR had been reached. Progression was defined as a local recurrence or appearance of new lesions. Follow-up evaluations were started 4 weeks after the date of the last SBRT treatment, and performed every 2–3 months for the first 2 year and every 6 months thereafter.

Toxicities were evaluated and graded according to the National Cancer Institute Common Toxicity Criteria Adverse Event version 3.0 (CTC AE v3.0) [20]. A diagnosis of radiation pneumonitis (RP) was made based on clinical symptoms (including cough, shortness of breath and fever), and radiologic findings in the absence of any other likely cause.

### Statistical methods

Statistical analyses were performed using SPSS software (version 17.0). The timing of recurrence or distant metastasis was recorded as the time at which the first image (FDG-PET or CT) showed abnormalities. Progression-free survival (PFS) time was measured from the date of the last SBRT to the date of the disease progression, and the overall survival (OS) time was considered from the last date of treatment to the date of analysis or date of loss from follow-up for patients alive. Patients without local recurrence or progression who discontinued the follow-up for any reason were censored on the date on the last tumor assessment.

The rates of PFS and OS curves were calculated using Kaplan-Meier analysis. Spearman’s rank correlation analysis was applied to determine correlations between the dose-volume histogram (DVH) -based parameters and the incidence of RP. Receiver-operating characteristics (ROC) curve analysis for each parameter was also applied to select the most relevant threshold to predict RP for grade 2 or higher. The optimal threshold for each DVH-based parameter was defined as the point yielding the minimal value for (1-sensitivity)^2^ + (1-specificity)^2^, according to the report from Akobeng [21]. A value of *p* < 0.05 was considered to have statistical significance.

## Results

The basic and clinical characteristics of the studied population are summarized in Table 2. The median age of the patients was 58 years (range: 45–74 years); most of them were male and with Eastern Cooperative Oncology Group (ECOG) performance status score 0–1 (21/23; 91.3%). Of the 23 patients, 10 (43.5%) had squamous-cell lung cancer, 10 had non-squamous cell lung cancer and three (13%) patients had, other pathological types respectively. According to our records, 9, 3, 5 and 4 patients had received resection of right upper lobe, right lower lobe, left upper lobe, and left lower lobe respectively. Two patients had undergone left pneumonectomy. The pathologic stage confirmed with surgery(Staging system, American Joint Committee on Cancer, 6^th^ edition) [22] in the present study were 6 (26.1%) stage I, 9 (39.1%) stage II, and 8 (34.8%) stage III respectively. The ILMs of the contralateral (12 patients) and ipsilateral (11 patients) lung were observed. The median time from surgery to ILMs and follow-up time was 16.0 months (range: 4.0-75.0 months, only 1 patient was diagnosed with ILM 75 months after pneumonectomy) and 14.0 months (range: 6.0-47.0 months), respectively.

**Table 2:** **Basic and clinical characteristics of the patients in present study (n = 23)**

Characteristics	Number of patients (%)
***Age (years)***	
Median (range)	58 (45–74)
***Gender***	
Male/Female	17 (73.9)/6 (26.1)
***ECOG*** ^***a***^ ***performance status***	
0-1	21 (91.3)
2	2 (8.7)
***Pathology of the primary tumor***	
Squamous-cell lung cancer	10 (43.5)
Non-squamous cell lung cancer	10 (43.5)
Sarcomatoid carcinoma	2 (8.7)
Small cell lung cancer	1 (4.3)
***Surgical method***	
Right upper lung lobectomy	9 (39.1)
Right lower lung lobectomy	3 (13.0)
Left upper lung lobectomy	5 (21.7)
Left lower lung lobectomy	4 (17.4)
Left pneumonectomy	2 (8.7)
***T staging after surgery*** ^***b***^	
T1/T2/T3/T4	3 (13.0)/11 (47.8)/8 (34.8)/1(4.3)
***N staging after surgery*** ^***b***^	
N0/N1/N2	10 (43.5)/6 (26.1)/7 (30.4)
***Tumor stage after surgery*** ^***b***^	
I	6 (26.1)
II	9 (39.1)
III	8 (34.8)
***Time from surgery to lung metastasis (months)***	
Median (range)	16.0 (4.0-75.0)
***Sites of lung metastasis***	
Contralateral lung of the primary tumor	12 (52.2)
Ipsilateral lung of the primary tumor	11 (47.8)
***Follow-up time since diagnosis of lung metastasis (months)***	
Median (range)	14.0 (6.0-47.0)

^*a*^: Eastern Cooperative Oncology Group; ^*b*^: staging system, 6^th^ edition, American Joint Committee on Cancer, 2002.

### Treatment

In the present study, 11 (47.8%), nine (39.1%) and three (13.0%) patients had received the prescription dose of 48 Gy (4 fractions), 50 Gy (5 fractions) and 56 Gy (7 fractions), respectively (Table 1). The median PTV was 48.4 cm^3^ (range: 26.0-110.2 cm^3^). The median lung volume was 2301.4 cm^3^ (range: 1983.4-2950.5 cm^3^). All patients underwent ABC and cone-beam CT guidance during treatment. Fourteen (60.9%) and 1(4.3%) patients received sequential and concurrent chemotherapy respectively, as parts of the treatment strategies. Three patients (13.0%) received tyrosine kinase inhibitors as the systematic treatment, and only five (21.7%) patients had not received systematic therapy.

### Local control and survival

**Figure 1:**
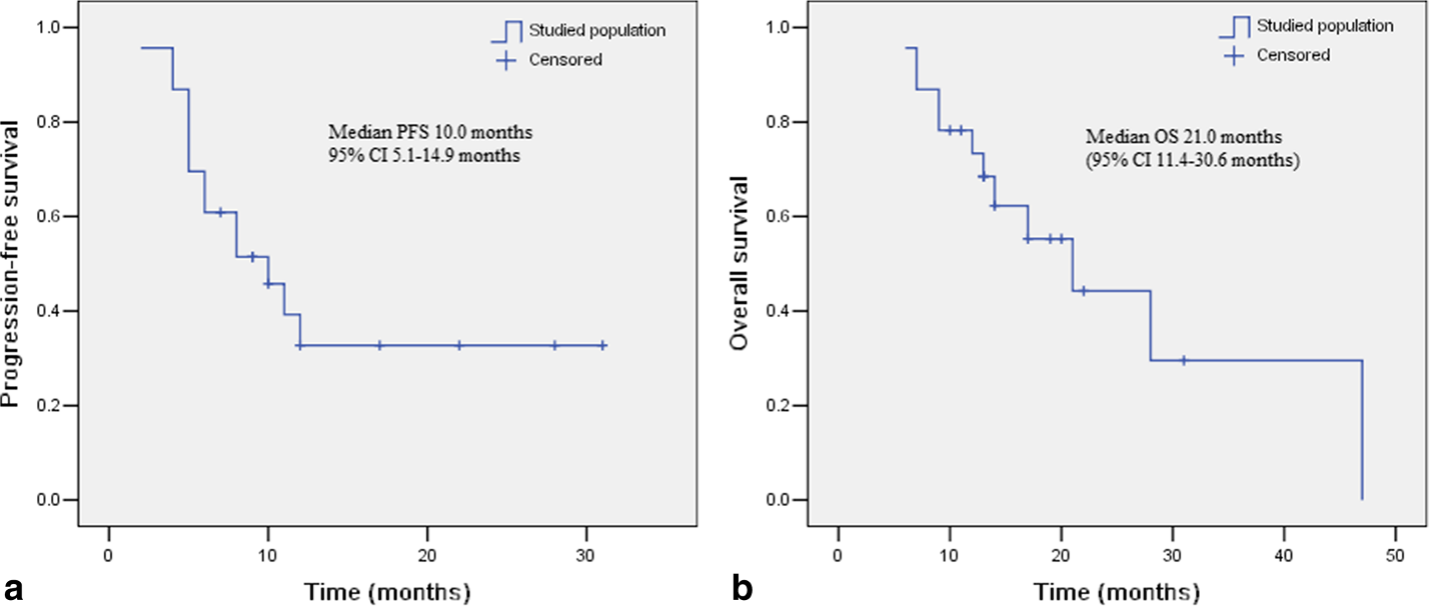
**Kaplan-Meier analysis of progression-free survival (a) and overall survival (b) in the present study.**

Follow-up studies continued until December 2013, with no patients lost to follow-up. Local recurrences were observed in two patients during follow-up, and the 1-year local control rate (LCR) was 91.3%. As Figure 1 shows, the median PFS and OS for the studied cohort were 10.0 months [95% confidence interval (CI) 5.1-14.9 months] and 21.0 months (95% CI 11.4-30.6 months), respectively.

**Figure 2:**
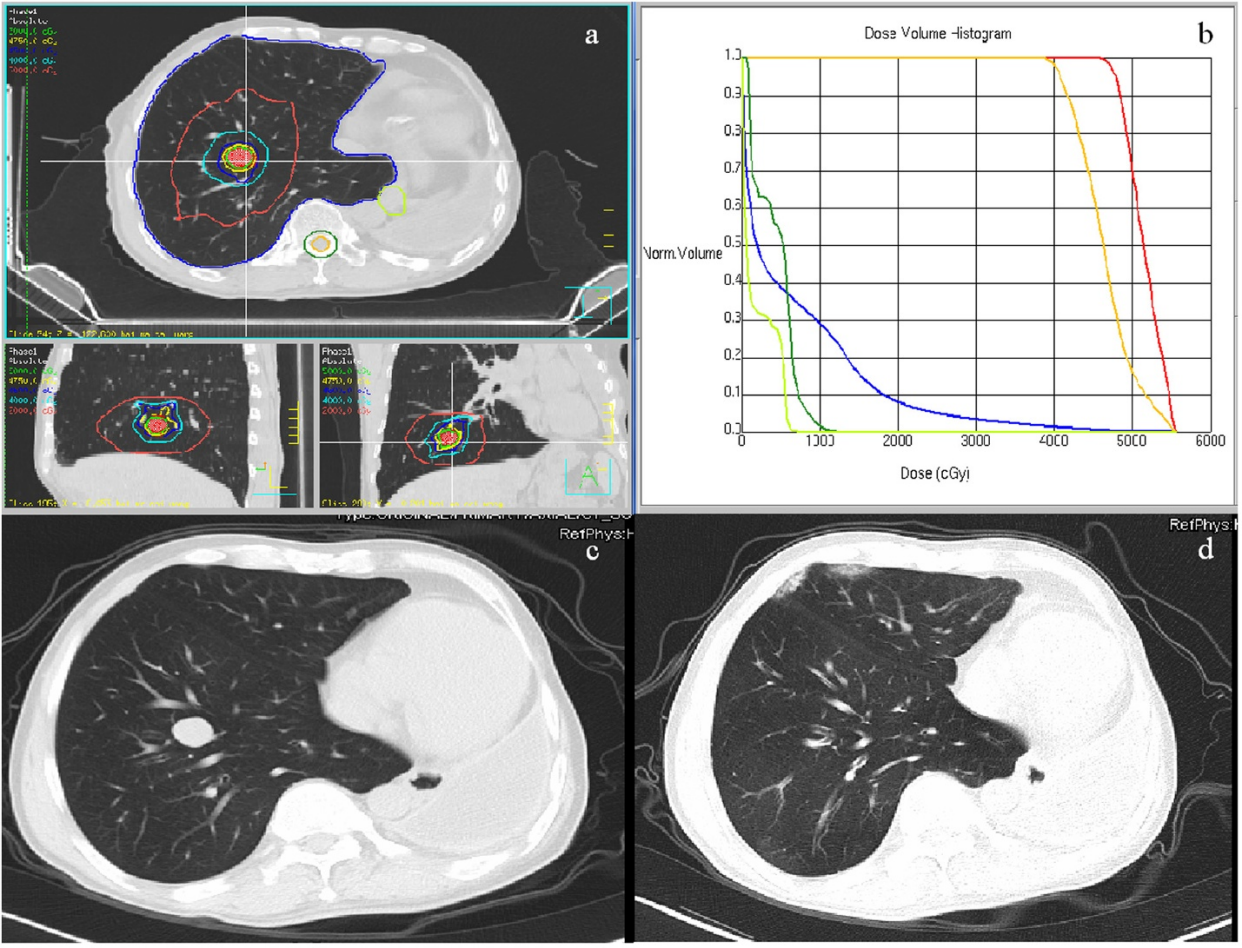
**Complete response after SBRT in the representative patient.** (**a:** irradiation isodose curves of the SBRT plan 50 Gy in 5 fractions; **b:** dose-volume histogram of the SBRT plan; **c:** CT image before SBRT; **d:** CT image 9 months after SBRT).

Figure 2 shows a patient with an ILM on the right pulmonary lobe, whose primary tumor was sarcomatoid carcinoma and who had received left pneumonectomy. The irradiation dose delivered was 10 Gy per fraction for five fractions. Figure 2 also shows a CR achieved 9 months after SBRT treatment (Figure 2c, d). Only light patchy shadows near the chest wall were observed as side effects of treatment.

### Treatment-related toxicities

All patients were evaluated for treatment-related toxicities (Table 3). The SBRT for ILM after pulmonary lobectomy was judged to be tolerable. The most common toxicity was cough (60.9%, 14 patients). Coughs of grade 2, 3, and 4 were recorded in four (17.4%), two (8.7%) and one (4.3%) patients, respectively. Other toxicities included shortness of breath (8.7%, two patients), acute esophagitis (8.7%, two patients) and chest wall pain (4.3%, one patient). No grade 5 toxicity was recorded.

**Table 3:** **The acute SBRT** ^***a***^ **-related toxicities in present study (n = 23)**

Toxicities ^***b***^	Toxicity grades, n (%)				
	Grade 0	Grade 1	Grade 2	Grade 3	Grade 4
***Radiation pneumonitis***					
Cough	9 (39.1)	7 (30.4)	4 (17.4)	2 (8.7)	1 (4.3)^*c*^
Shortness of breath	21 (91.3)	0	1 (4.3)	0	1 (4.3)^*c*^
***Other treatment-related toxicities***					
Chest wall pain	22 (95.7)	0	1 (4.3)	0	0
Acute esophagitis	21 (91.3)	0	2 (8.7)	0	0

^*a*^: stereotactic body radiation therapy; ^*b*^ : according to the Common Toxicity Criteria for Adverse Events, version 3.0; ^*c*^: same patient.

**Figure 3:**
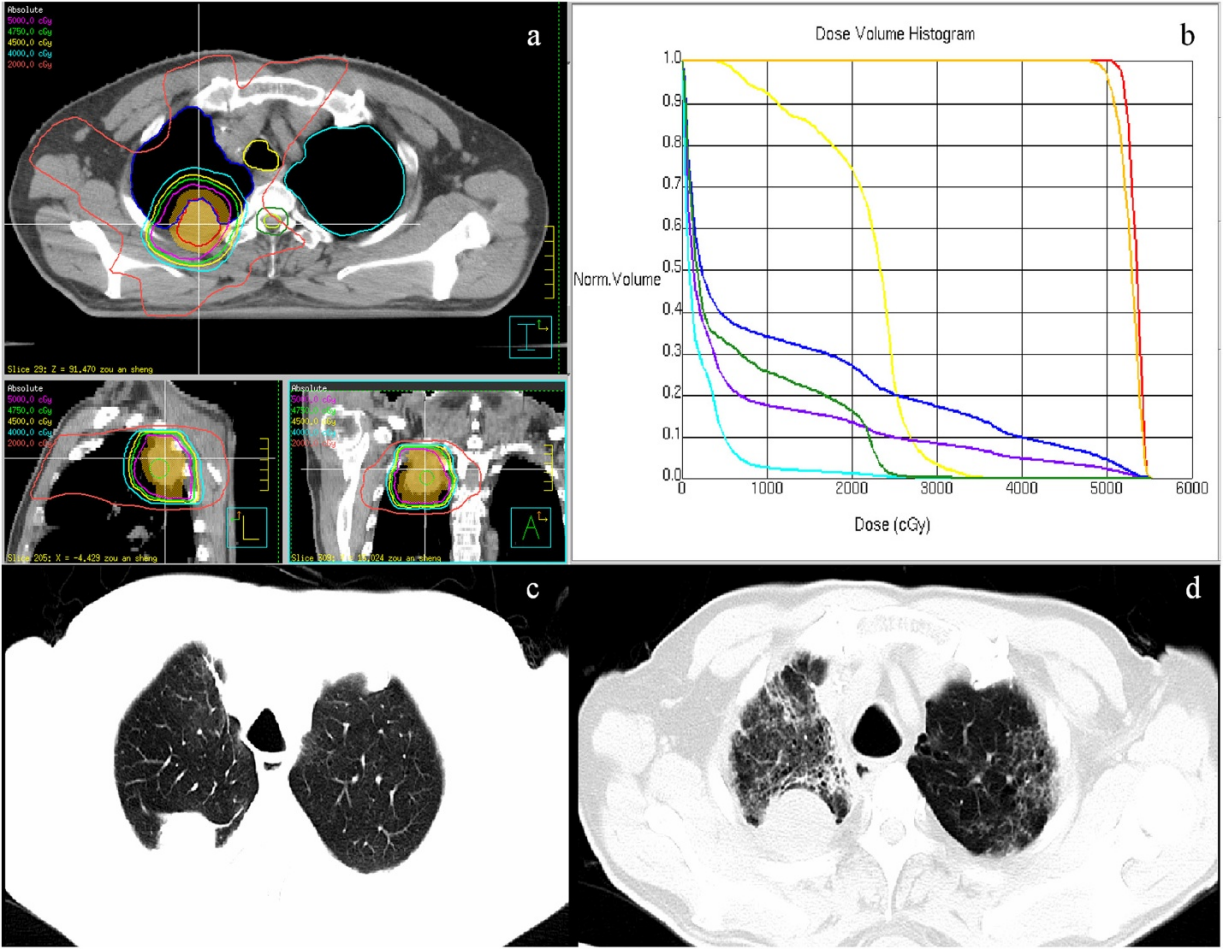
**Representative patient who developed grade 4 radiation pneumonitis.** (**a**: irradiation isodose curves of the SBRT plan 50 Gy in 5 fractions; **b**: dose-volume histogram of the SBRT plan, arrow pointing the curve of the total lung; **c**: CT image before SBRT; **d**: CT image one month after SBRT).

Figure 3 shows a patient with ILM of small-cell lung cancer on the right upper lobe after right lower lobectomy. The prescription dose was 10 Gy per fraction for five fractions. The volume of lung exposed to a minimum dose of 20 Gy (V_20_) and V_30_ of total lungs was less than 15 and 10% respectively. One month after SBRT, the patient experienced severe cough and dyspnea; CT scans showed a stable disease as the response to treatment, and widespread, patchy shadows on both upper lobes (grade 4 RP). After steroid therapy for 6 weeks, the patient recovered.

### Correlations between lung parameters and incidence of RP

Table 4 summarizes the correlations between the DVH-based lung parameters and acute RP of grade 2or higher. The incidence of acute RP of grade ≥2 was significantly associated with the PTV (mean: 59.0*vs.*45.0 cm^3^, *p* = 0.039). Another possible predictive parameter was the V_5_ of the ipsilateral lungs (IpV_5_) (mean: 51.0 *vs.* 44.0%, *p* = 0.034). Other lung parameters did not significantly correlate with the incidence of acute RP of grade 2 or higher.

**Table 4:** **Correlations between the DVH** ^***a***^ **-based parameters and acute grade ≥2 RP** ^***b***^ **in present study (n = 23)**

	Grade ≥2 RIP (n = 8)	Grade 0–1 RIP (n = 15)	***p***value
	mean ± SD	mean ± SD	
***Total Lungs***			
V_5_ (%)^*c*^	36.0 ± 6.0	35.0 ± 4.0	0.141
V_10_ (%)^*c*^	16.0 ± 4.0	14.0 ± 2.0	0.125
V_20_ (%)^*c*^	4.8 ± 2.0	4.4 ± 1.2	0.696
V_30_ (%)^*c*^	2.0 ± 1.0	2.0 ± 1.0	0.990
MLD (Gy) ^*d*^	3.8 ± 1.6	3.7 ± 1.3	0.077
***Contralateral lungs***			
V_5_ (%)	26.0 ± 7.0	25.0 ± 9.0	0.421
V_10_ (%)	18.0 ± 7.0	16.0 ± 9.0	0.277
V_20_ (%)	3.2 ± 1.1	2.4 ± 0.9	0.210
V_30_ (%)	1.6 ± 0.5	1.6 ± 0.6	0.992
MLD (Gy)	2.8 ± 0.6	2.6 ± 0.2	0.483
***Ipsilateral lungs***			
V_5_ (%)	51.0 ± 9.1	44.0 ± 4.0	0.034
V_10_ (%)	22.0 ± 6.0	19.0 ± 4.0	0.273
V_20_ (%)	6.0 ± 2.8	5.2 ± 2.2	0.454
V_30_ (%)	2.6 ± 1.0	2.8 ± 1.1	0.824
MLD (Gy)	5.9 ± 1.3	5.7 ± 1.1	0.290
***PTV volume (cm*** ^***3***^ ***)***	59.0 ± 9.6	45.0 ± 9.9	0.039

^*a*^: dose-volume histogram; ^*b*^: radiation pneumonitis; ^*c*^: the percentage of the lung volume that received more than 5, 10, 20 and 30 Gy irradiation dose, respectively; ^*d*^: mean lung dose.

**Figure 4:**
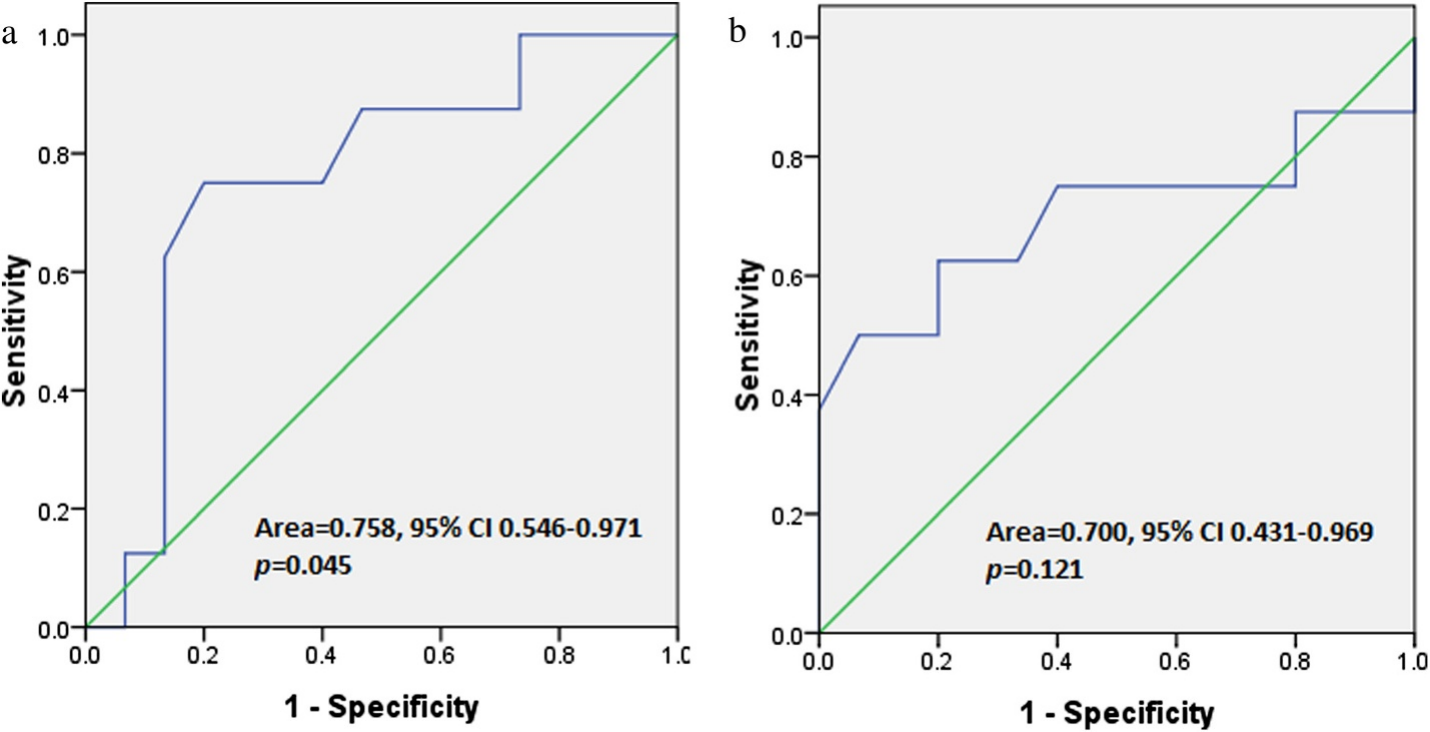
**Receiver operating characteristics (ROC) curve analysis in present study.** (**a**: for PTV volume and **b**: for V_5_ of the ipsilateral lung).

By ROC analysis, the areas under curve were 0.758 (*p* = 0.045) and 0.700 (*p* = 0.121) for PTV and IpV_5_, respectively (Figure 4). Additionally, the optimal values to predict acute RP of grade 2 or higher were 59 cm^3^ (for PTV) with sensitivity of 75% and specificity of 80.0% (Table 5). The optimal value for IpV_5_ was 51%, with sensitivity/specificity of 62.5% and 80.0%, respectively.

**Table 5:** **ROC** ^***a***^ **curve analysis for DVH** ^***b***^ **-based parameters related to acute grade ≥2 RP** ^***c***^ **in present study**

Parameters	Optimal threshold		
	Value	Sensitivity	Specificity
PTV volume (cm^3^)	59	75.0%	80.0%
Ipsilateral lung V_5_^*d*^ (%)	51	62.5%	80.0%

^*a*^: receiver operating characteristic; ^*b*^: dose-volume histogram; ^*c*^: radiation pneumonitis; ^*d*^: the percentage of the ipsilateral lung volume that received more than 5 Gy irradiation dose.

## Discussion

The treatment of cancer patients with ILM is a common clinical problem. SBRT is an appealing treatment option, but little is known about its use in the post-lobectomy setting. Our initial experience of using hypo-fractionated SBRT for ILM after pulmonary lobectomy is presented here for the first time.

The 1-year LCR was 91.3% for all patients in the present study, the median PFS and OS were 10.0 and 21.0 months, respectively. Our data are consistent with those of other reports on SBRT for metastatic lung cancer [23–25], especially the report from Norihisa *et al.* whose prescription dose was 48–60 Gy/4-5 fractions and LCR 90.0% [25]. These clinical outcomes were comparable with those achieved by surgical metastasectomy [6]. In 2009, Rusthoven *et al.* reported a prospective multi-institutional phase I/II trial of SBRT for metastatic lung tumor, and reported actuarial LCRs at 1 and 2 years after SBRT of 100 and 96%, respectively. After a median follow-up of 15.4 months, a median survival of 19 months was achieved using a prescription dose of 48–60 Gy in three fractions [26]. Our data also confirmed that the main pattern of failure after SBRT was distant metastasis, as was concluded in a systematic review by Chi *et al.* [27].

A few studies have evaluated the outcomes of SBRT among patients after pneumonectomy [28–30]. Authors from the VU University Medical Center in the Netherlands reported on 15 patients with a second primary lung cancer who received SBRT after pneumonectomy in 2009 [28]. After a median follow-up time of 16.5 months, no local failures were observed and the 1-year actuarial disease-free survival rate was 92%. In 2013, the same investigatorsy updated their data and compared the outcomes between SBRT, hypo-fractionated radiotherapy, and conventional radiotherapy among such patients [29]. In this paper, they reported a median OS of 39 months during follow-up. Thompson *et al*. identified 13 patients with newly identified lung malignancy after surgery from 406 patients who received SBRT [30]. The doses delivered were 60 Gy/3 fractions (n = 1), 54 Gy/3 fractions (n = 1), 48 Gy/4 fractions (n = 7), 60 Gy/8 fractions (n = 2), and 50 Gy/10 fractions (n = 3). The Median survival was 29 months, and no local failures were observed. In our cohort, the targets were metastatic tumors; thus, even though the local control rate was similar to that the reported in the aforementioned studies discussed above, the OS (median 21 months) among our patients was shorter than in those patients with newly diagnosed lung cancer.

The SBRT treatment was well tolerated in our patient population. The most common toxicity was cough (60.9%), and acute RP of grade 3 or worse was observed in three patients (13.0%). Other treatment-related toxicities included dyspnea, chest wall pain, and acute esophagitis. These findings are consistent with the reports evaluating SBRT in newly identified lung cancer after pneumonectomy [28, 30] and in medically inoperable or operable NSCLC [26, 31–37]. In the post-pneumonectomy settings, Haasbeek *et al*. reported that only two2 patients experienced toxicity of grade 3 or higher toxicity [28]. In a report by Thompson *et al.*, two2 patients in a 13-patient cohort had grade 3 RP [30]. For medically inoperable or operable NSCLC, in the RTOG trial 0236, with a prescription dose of 54 Gy in three fractions, treatment-related grade 3 and 4 toxicities of pulmonary or upper respiratory tract were observed in 14.5 and 1.8% of patients, respectively [31]. In a phase II study of SBRT, Baumann *et al.* reported that grade 3 pulmonary toxicities were seen in 11.8 and 12.5% of patients in the cardio-vascular disease group and chronic obstructive pulmonary disease group, respectively [32]. In a study from Japan (JCOG 0403), Nagata *et al.* reported grade 3 toxicity in 6.2% of their patients who received SBRT treatment [33]. For metastatic lung cancer, Rusthoven *et al.* reported that grade 2 RP occurred in only one patient (2.6%) in their multi-institutional phase I/II trial [26]. The investigators suggested that the low rate of pneumonitis observed might have contributed to the dose constraint used (V_15_ < 35%) in their patient population. However this needs to be confirmed in a larger cohort of patients because in our study, one patient developed grade 4 RP, and the V_15_ was less than 17% according to the DVH analysis.

Several studies have evaluated the potential value of the DVH-based lung parameters in predicting acute “symptomatic” RP after SBRT [34–38]. The RP rates were reported within a range of 9.4% to 28.0%, and the possible predictive factors for RP differed among these studies. In 2007, Yamashita *et al.* reported that 29% of their patients had developed grade 2 or worse RP after SBRT (48 Gy in four fractions), and that the conformity index was the only factor associated with incidence of RP [34]. Ricardi *et al.* observed a good correlation between mean lung dose (MLD) and grade 2–3 pulmonary toxicity (*p* = 0.008, odds ratio 1.5) in a 60-patients cohort after SBRT of 15 Gy per fraction × 3 fractions [35]. Moreover, reports by Borst *et al.*, Guckenberger *et al.* and Barriger *et al*. indicated that MLD (ipsilateral or total lung) was correlated with incidences of symptomatic RP after pulmonary SBRT [36–38]. Onestudy indicated that V_5_ of total lung >37% and V_5_ of contralateral lung > 26% were suitable predictors of pneumonitis in a cohort of patients treated with SBRT [39]. Additionally, Guckenberger *et al.* reported that the V_2.5_-V_50_ were correlated with incidences of RP with a continuous decrease of the goodness of fit for higher doses [37]. In a Japanese study, Matsuo *et al.* concluded that the symptomatic RP rate was significantly lower in the group with PTV < 37.7 mL compared with the group with larger PTV (11.1 vs. 34.5%, *p* = 0.02) [40]. In the present study, we also identified two factors that might significantly be associated with RP of grade 3 or worse after SBRT in the post-lobectomy situation: PTV and IpV_5_. Like other parameters already mentioned, the value of these two factors as the thresholds in SBRT for ILM warrants further clinical investigations.

To the best of our knowledge, there is little information regarding the correlation between various DVH-based factors and lung toxicity in radiotherapy among patients after pulmonary lobectomy. Uno *et al.* reported that higher in V_13/20_ and MLD values could be a surrogate for RP in NSCLC patients after lobectomy [41]. While the treatment was concurrent chemo-radiotherapy for recurrent NSCLC, these parameters could not be easily followed in an SBRT setting.

Some limitations of the present study justify mention. First, this analysis was retrospective and the number of patients evaluated was limited, thus leading to a bias of selection. Second, being a multicenter study, there was no central data review, and the determination of RP can be subjective and challenging. Third, there is an obvious difference between the RTOG system, CTC AE v2.0, and CTC AE v3.0 regarding steroid use for RP. Tucker *et al.* reported 442 patients who received definitive radiotherapy using these three toxicity grading systems: RP of grade 2 or worse was observed in 29, 25, and 44% of patients according to RTOG, CTC AE v2.0 and CTC AE v3.0, respectively [42]. Therefore, attention should be paid to the toxicity grading systems when interpreting the results discussed herein.

## Conclusions

In conclusion, our results indicate that SBRT is a promising tool for the salvage treatment of ILM in patients who had previously received pulmonary lobectomy. PTV and IpV_5_ are possible predictive factors for the development of symptomatic RP. Prospective studies are needed to verify these findings.
